# Association of body mass index and visceral fat with aortic valve calcification and mortality after transcatheter aortic valve replacement: the obesity paradox in severe aortic stenosis

**DOI:** 10.1186/s13098-017-0285-2

**Published:** 2017-10-19

**Authors:** Jennifer Mancio, Paulo Fonseca, Bruno Figueiredo, Wilson Ferreira, Monica Carvalho, Nuno Ferreira, Pedro Braga, Alberto Rodrigues, Antonio Barros, Ines Falcao-Pires, Adelino Leite-Moreira, Vasco Gama Ribeiro, Nuno Bettencourt

**Affiliations:** 1 Department of Cardiothoracic Surgery and Physiology, Faculty of Medicine, University of Porto, Alameda Professor Hernani Monteiro, 4200-319 Porto, Portugal; 2 Department of Cardiology at the Centro Hospitalar de Vila Nova de Gaia e Espinho, Vila Nova de Gaia, Portugal; 3 Department of Cardiothoracic Surgery, Centro Hospitalar de Sao Joao, Porto, Portugal

**Keywords:** Severe aortic stenosis, Aortic valve calcification, Transcatheter aortic valve implantation, Obesity, Body mass index, Visceral abdominal fat, Mortality, Cardiac computed tomography

## Abstract

**Background:**

Previous studies showed that metabolic syndrome is associated with aortic valve calcification (AVC) and poor outcomes in aortic stenosis (AS). However, if these associations change and how body fat impacts the prognosis of patients in late stage of the disease have been not yet explored.

**Aims:**

To determine the association of body mass index (BMI) and visceral fat with AVC and mortality after transcatheter aortic valve replacement (TAVR).

**Methods:**

This was a prospective cohort of 170 severe AS patients referred to TAVR. We quantified AVC mass score and fat depots including epicardial adipose tissue, intrathoracic fat, and abdominal visceral (VAF) and subcutaneous fats by computed tomography. Fat depots were indexed to body surface area. All-cause and cardiovascular-related deaths after TAVR were recorded over a median follow-up of 1.2 years.

**Results:**

Higher AVC mass was independently associated with low BMI and low VAF. All-cause mortality risk increased with the decrease of BMI and increment of VAF. A stratified analysis by obesity showed that in non-obese, VAF was inversely associated with mortality, whereas in obese, high VAF was associated with higher mortality (p value for interaction < 0.05). At long-term, hazard ratio [HR] with non-obese/low VAF was 2.3 (95% confidence interval [CI] 1.1–4.9; p = 0.021) and HR with obese/high VAF was 2.5 (95% CI 1.1–5.8; p = 0.031) compared with obese/low VAF patients.

**Conclusions:**

In AS patients submitted to TAVR, BMI and VAF were inversely associated with AVC. Pre-intervention assessment of VAF by computed tomography may provide a better discrimination of mortality than BMI alone.

**Electronic supplementary material:**

The online version of this article (doi:10.1186/s13098-017-0285-2) contains supplementary material, which is available to authorized users.

## Background

Obesity, defined through body mass index (BMI), is associated with clustering of other cardiovascular risk factors. These factors have been associated with the development of aortic stenosis (AS) and were associated with the incidence of cardiovascular events both in general and AS populations [1–3]. In the Multi-Ethnic Study of Atherosclerosis (MESA) prospective cohort, metabolic syndrome was associated with a significant increase in incident aortic valve calcification (AVC) [4]. Similarly, the ASTRONOMER (Aortic Stenosis Progression Observation Measuring Effects of Rosuvastatin) study showed that metabolic syndrome was associated with increased progression rate of AS, as well as abnormal left ventricular geometry and presence of left ventricular myocardial dysfunction [5, 6].

However, better survival in overweight and mildly obese patients compared with the normal weight has been reported after surgical aortic valve replacement [7–9], and similar data were reported among transcatheter aortic valve replacement (TAVR) [10]. This phenomenon, called the “Obesity Paradox”, was described in other types of populations including diabetes, hypertension, heart failure, established coronary artery disease and peripheral arterial disease [11–13]. Despite the clinical evidence suggesting an obesity paradox, this association may simply reflects BMI inherent limitations as a predictor of mortality in populations who already have the disease, particularly in the elderly [14], since ageing is associated with significant weight loss and body fat redistribution [15, 16].

Therefore, in the present study we aimed to determine the associations of global (BMI), and regional (intra-thoracic fat, epicardial fat and abdominal visceral and subcutaneous fat) adiposities with AVC, and with the incidence of all-cause and cardiovascular disease-related deaths in a cohort of symptomatic severe AS patients following TAVR.

## Methods

### Patient population

This was a prospective cohort of symptomatic severe AS patients referred to TAVR at our institution between August 2007 and November 2015. All patients were recruited as part of the institutional registry of patients submitted to TAVR. The study protocol was approved by the Institutional Ethical Committee, and all patients provided written informed consent. Before intervention, these patients were assessed by a multidisciplinary Heart Team according to a pre-specified protocol that includes anthropometric, clinical, echocardiographic (transthoracic and transoesophageal), computed tomographic angiography and invasive coronary angiography evaluations. Comorbidities including New York Heart Association class, arterial hypertension, dyslipidemia, diabetes, smoking status, chronic obstructive pulmonary disease, stroke, peripheral arterial disease, history of cancer, as well as ongoing medication, were collected. BMI was calculated on the basis of direct measurements as the weight divided by the square of height; normal weight, overweigh and obese categories was defined on the basis of World Health Organization definition as BMI < 25, 25–30 or ≥ 30 kg/m^2^, respectively [17]. The intervention risk of morbi-mortality was estimated using the risk prediction models developed by the Society of Thoracic Surgeons (STS) and by the European Association of Cardio-thoracic Surgeons (Euroscore II) [18].

### Computed tomography protocol scan

Computed tomography (Somatom Sensation Cardiac 64, Siemens, Forchheim, Germany) was performed 1–3 months before the procedure. The exam included two different acquisitions: the first for abdominal fat quantification, and the second for aorta, iliac and femoral arteries angiography. At the beginning of the exam, an abdominal single slice acquisition was performed between L4 and L5 as originally described by Borkan et al. [19]; the radiographic factors were 120 kV and 216 mAs with 5 mm thickness resulting in an estimated radiation exposure of 0.06 mSv. Subsequently, an helical acquisition to include the whole aorta and iliofemoral arteries was performed with a tube voltage of 100 kV, tube current modified according to patient size, a rotation time of 330 ms, a slice collimation of 0.6 mm, and a pitch of 1.1; images were reconstructed at a slice thickness of 0.75 mm and patients received an intravenous injection of 80–100 mL of contrast agent (Ultravist 370, BayerSchering Pharma, Berlin, Germany), followed by a 40 mL flush of normal saline solution, both at flow rates of 4.5 mL/s; luminal attenuation was assessed by measuring the mean signal value (Hounsfield Unit, HU) in the aortic root.

### Aortic valve calcium mass quantification

Using the same sets of images acquired for aorta angiography, the non-coronary; right-coronary and left-coronary cusps calcium mass scores were estimated using a dedicated software (3mensioValves™) with threshold of 650 HU for calcium detection, as previously described [20] subsequently, total AVC mass score was derived by the sum of individual cusp AVC mass scores.

### Thoracic and abdominal fat analyses

Fat analyses was performed in a dedicated workstation (SyngoVolume, Siemens Medical Solutions) using a predefined image display setting [window with, − 150 to − 50 HU] to identify voxels that correspond to adipose tissue. Thoracic fat including epicardial adipose tissue and intrathoracic fat volumes were assessed using the thoracic aorta angiography imaging; for epicardial fat volume analysis, the pericardium was manually traced for every 10 mm from the right pulmonary artery to the diaphragm to determine a region of interest; total Intrathoracic fat volume was defined as any adipose tissue located within the thorax from the level of the first costal arch to the diaphragm and from the chest wall to the descending aorta. Abdominal fat analyses was obtained from the abdominal acquisition: total abdominal fat area was the sum of adipose tissue presented in the examined abdominal slice, and a cursor pointer was used to trace the visceral abdominal fat (VAF) area by delineating the abdominal wall muscular layer; the subcutaneous abdominal fat (SAF) was obtained by subtracting VAF from total abdominal fat. All body fat depots were normalized to body surface area. Intra- and interrater reliabilities for each fat measurement were evaluated in 55 random patients; the intraclass correlation coefficients’ are shown in Additional file 1: Table S1.

### Follow-up

Survival status was obtained by clinical consultation and by phone contact with patients, their relatives or their physician, as well as, by consultation of national records of deaths. Cause of death was categorized as cardiovascular or non-cardiovascular. Cardiovascular mortality was defined as death attributable to congestive heart failure, myocardial infarction, or sudden death. For this analysis we excluded three patients who died during TAVR or in the first day after the procedure (aorta ring rupture = 1, ventricular fibrillation = 1, hemorrhagic stroke = 1).

### Statistical analysis

Continuous variables are reported as mean ± SD for normally distributed data or median (Q2) and 25th and 75th quartiles (Q1 and Q3, respectively) for non-normally distributed data. Discrete variables are given in frequency. Differences between obesity-groups, considering normal weight as reference, were compared using T test for normally distributed and Mann–Whitney U Test for non-normal distributed-continuous variables; Chi square test or Fisher-exact test were used to compare categorical variables. Considering the skewed distribution of AVC mass score, we determine the Spearmen Rho correlation coefficients between AVC mass and body fat, and the AVC mass square root was calculated to graphically represent its correlation with body fat. Median regression analysis was performed to modeling the association between AVC mass and body fat; after analyzing body fat as the sole predictor, we further tested the following models: Model 1: age and sex-adjusted; Model 2: age, sex and BMI-adjusted; Model 3: model 2 + arterial hypertension, systolic blood pressure, diastolic blood pressure, diabetes and dyslipidemia. To study if the association between body fat and AVC differed according to the severity of the disease, we performed a stratified analysis by age-, and STS-morbimortality score-groups. Survival analysis was performed using Cox proportional hazards models and Kaplan–Meier curves compared by the log-rank test, while the proportional hazards assumption was tested and confirmed by means of Schoenfeld residuals. The predictive value of BMI and VAF for the endpoints of all-cause and cardiovascular mortality was first tested in univariate Cox regression analysis using fat measures both as continuous and categorical predictors (VAF categories defined according to the median value as low VAF if VAF index < 74.2 and high VAF if VAF index ≥ 74.2 cm^2^/m^2^). The association of BMI and VAF with mortality was also tested by applying the fractional polynomials method to model non-linear relationships. The best-fitting fractional polynomial model was chosen and the results are graphically presented. Given the apparent different association between VAF and mortality according to BMI-categories, we then combined BMI-, and VAF-groups and tested if the association of VAF with mortality differed according to obesity in a stratified analysis by BMI-categories. Multivariate adjustment included sex, age, systolic blood pressure, diastolic blood pressure, arterial hypertension, dyslipidemia, diabetes mellitus, smoking status, peripheral arterial disease, chronic obstructive pulmonary disease, neoplasia, atrial fibrillation, New York Heart Association class, mean aortic valve gradient, maximum aortic valve gradient, left ventricular ejection fraction. All analysis was performed using STATA software (version 13.1, StataCorp LP, Texas, US). All tests were two-sided and α was set at 0.05.

## Results

Overall, 170 patients with symptomatic severe AS undergoing TAVR (mean age 79 ± 7.5 years; 49.4% of male) were included in this analysis which detailed characteristics by BMI are showed in Table 1. No significant differences were found between BMI-groups except for the higher frequency of atrial fibrillation in the obese compared with the normal weight.

**Table 1:** Baseline characteristics by BMI categories

	Overall n = 170	Normal weight n = 64	Overweight n = 62	Obese n = 44	p value^a^	p value^b^
Demographics						
Age, years	78.9 ± 7.5	79.3 ± 8.3	79.5 ± 7.1	77.6 ± 6.8	1	0.08
Male, n (%)	84 (49.4)	40 (62.5)	29 (46.8)	15 (24.0)	0.07	0.19
Risk factors						
Hypertension, n (%)	138 (81.2)	48 (75.1)	50 (80.7)	40 (90.9)	0.44	0.15
Hyperlipidemia, n (%)	118 (69.4)	38 (59.4)	44 (70.2)	36 (81.8)	0.17	0.20
Diabetes mellitus, n (%)	70 (41.2)	21 (32.8)	25 (40.3)	24 (54.6)	0.38	0.15
Current smoker, n (%)	5 (2.9)	2 (3.1)	1 (1.6)	2 (4.5)	0.08	0.85
Former smoker, n (%)	23 (13.5)	8 (12.5)	9 (14.5)	6 (13.6)	0.87	0.79
History/comorbidity						
AF, n (%)	57 (33.5)	23 (35.9)	15 (24.2)	19 (43.2)	0.15	0.04
COPD, n (%)	49 (28.8)	19 (36.7)	15 (24.2)	15 (34.1)	0.48	0.27
Anaemia, n (%)	75 (44.1)	25 (39.1)	28 (45.2)	22 (50)	0.48	0.26
Neoplasia, n (%)	23 (13.5)	10 (15.6)	10 (16.1)	3 (6.8)	0.94	0.18
Echocardiography						
AVA, cm^2^	0.6 ± 0.2	0.58 ± 0.14	0.63 ± 0.17	0.61 ± 0.22	0.62	1
AVAi, cm^2^/m^2^	0.3 ± 0.09	0.35 ± 0.07	0.35 ± 0.09	0.32 ± 0.11	0.94	0.06
PG, mmHg	82.3 ± 22.4	84.1 ± 21.6	78.1 ± 19.5	85.3 ± 26.3	0.14	0.61
MG, mmHg	50.2 ± 14.6	50.9 ± 13.9	47.7 ± 13.6	52.1 ± 16.7	0.23	1
LVEF, %	51.2 ± 10.4	49.3 ± 12.1	52.8 ± 9.6	52.5 ± 8.1	0.51	0.55
LA, mm	48 ± 8	48.9 ± 7.8	47.7 ± 6.8	50.2 ± 6.0	1	0.29
LVEDD, mm	52 (47–56)	52.8 ± 7.7	50.9 ± 7.5	52.5 ± 6.7	0.62	1
Coronary angiography						
≥ 1 vessel disease, n (%)	58 (51.3)	22 (46.8)	24 (61.5)	12 (44.4)	0.17	0.84
Computed tomography						
Body fat depots						
EATi, mL/m^2^	51.4 ± 21.6	44.8 ± 23.0	52.6 ± 18.1	59.1 ± 21.8	0.04	< 0.01
ITFi, mL	132.1 ± 54.0	110.8 ± 45.7	139.5 ± 56.7	151.1 ± 51.9	0.02	< 0.01
VAFi, cm^2^/m^2^	81.2 ± 36.8	59.9 ± 31.1	88.4 ± 31.3	100.6 ± 36.9	< 0.01	< 0.01
SAFi, cm^2^/m^2^	103.7 ± 43.5	77.4 ± 31.0	109.9 ± 41.1	130.2 ± 42.4	< 0.001	< 0.001
TAFi, cm^2^/m^2^	187.3 ± 71.5	136.8 ± 17.2	204.8 ± 71.5	230.8 ± 58.0	< 0.001	< 0.01
Aortic valve calcification						
Total mass score	533 (277; 961)	751 (344; 1144)	516 (283; 883)	456 (187; 908)	0.04	0.03
RC mass score	156 (77; 526)	151 (65; 398)	156 (83; 264)	168 (64; 301)	0.89	0.68
LC mass score	237 (118; 401)	279 (140; 472)	239 (134; 375)	171 (74; 284)	0.43	0.05
NC mass score	161 (77; 303)	197 (110; 342)	131 (81; 417)	127 (38; 239)	0.07	0.08
Deaths						
All-cause deaths, n (%)	49 (28.8)	21 (32.8)	15 (24.2)	13 (29.5)	0.28	0.72
Cardiovascular related-deaths, n (%)	25 (14.7)	10 (17.2)	7 (12.7)	8 (19.5)	0.50	0.77

Values are mean ± SD or n (%)

*AVA* aortic valve area, *AVAi* aortic valve area index, *AF* atrial fibrillation, *COPD* chronic obstructive pulmonary disease, *EATi* epicardial adipose tissue volume, *ITFi* intrathoracic fat volume index, *LA* left atrium, *LVEF* left ventricular ejection fraction, *LVEDD* left ventricular end-diastolic dimension, *MG* mean gradient, *PG* peak gradient, *SAFi* subcutaneous abdominal fat index, *TAFi* total abdominal fat index, *VAFi* visceral abdominal fat index

^a^Comparison between normal weight and overweight

^b^Comparison between normal weight and obese

### Body fat and AVC mass score

**Fig. 1:**
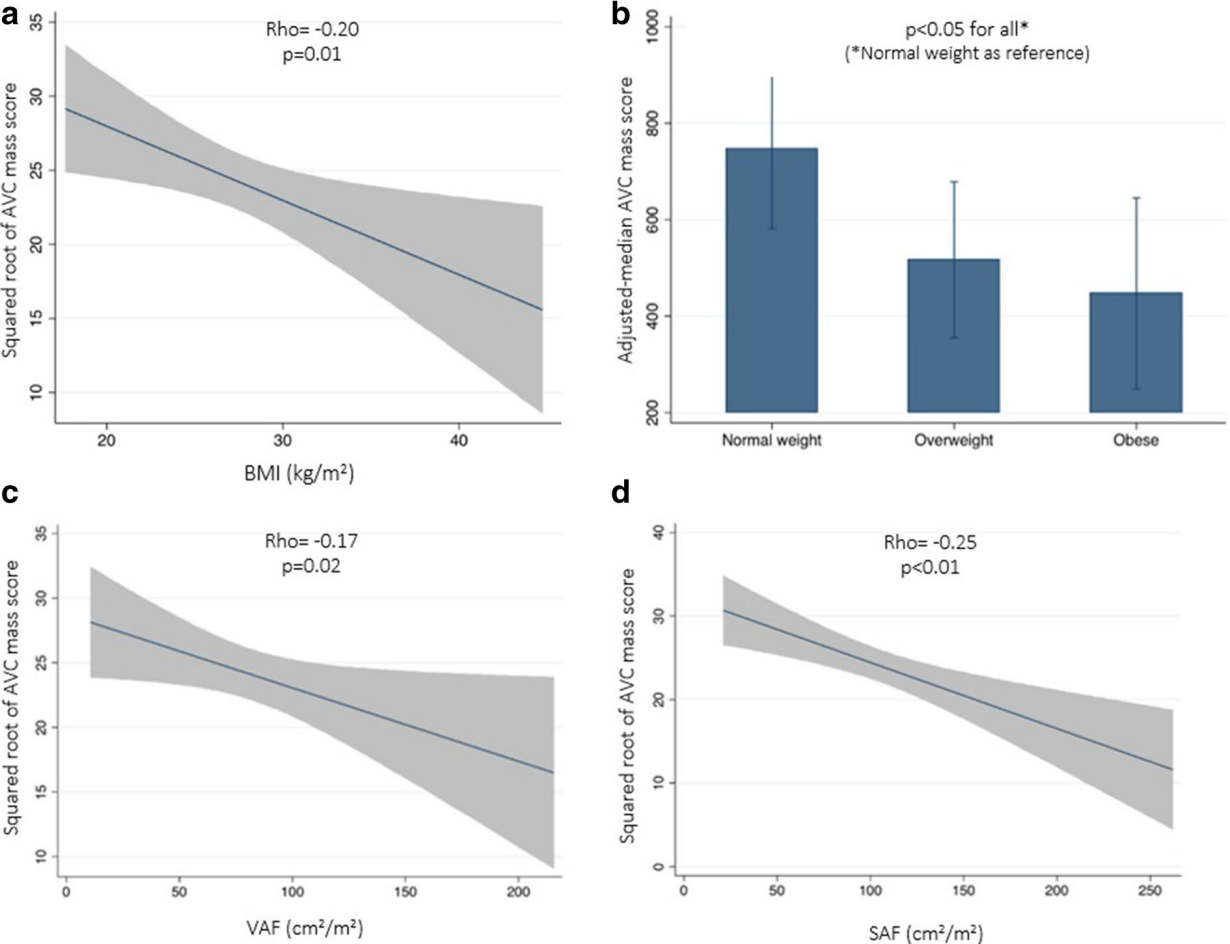
Association between body fat and AVC mass score. AVC was negatively correlated with BMI (**a**), VAF (**c**) and SAF (**d**) indices. The age, sex and cardiovascular risk factors adjusted-median of AVC (with 95% confidence interval) was higher in normal weight compared with the overweight and obese (**b**). *AVC* aortic valve calcification, *BMI* body mass index, *SAF* subcutaneous abdominal fat, *VAF* visceral abdominal fat

Table 2 shows the crude- and adjusted-associations between body fat measures and AVC mass score. Figure 1a–d illustrates the correlation between BMI and abdominal fat with the square root of AVC mass score. In univariate analysis, BMI, VAF and SAF were inversely associated with the median total AVC mass. After adjustment for age, sex and traditional cardiovascular risk factors, VAF remained inversely related to AVC mass, but not SAF. The adjusted-AVC mass score was significantly higher in normal weight as compared with overweight and obese patients. Thoracic fat depots (both epicardial and intrathoracic) were not associated with AVC mass.

**Table 2:** Univariate and multivariable median regression for the association between body fat with total AVC mass score

Body fat	Unadjusted			Age and sex-adjusted			MV-adjusted		
	B	(95% CI)	p value	B	(95% CI)	p value	B	(95% CI)	p value
BMI	− 27.1	(− 47.3: − 6.59)	0.01	− 20.2	(− 40.3; − 1.17)	0.03	− 23.1	(− 44.8: − 1.26)	0.02
Overweight^a^	− 76.7	(− 116.2; − 37.2)	< 0.0001	− 40.9	(− 76.3; − 5.4)	0.02	− 37.3	(− 72.8; 1.8)	0.04
Obese^a^	− 77.3	(− 120.6; − 33.9)	< 0.0001	− 42.3	(− 82.4; − 2.2)	0.03	− 36.9	(− 78.0; 4.3)	0.07
Obese^b^	− 0.59	(− 44.4; 43.1)	0.97	− 1.4	(− 41.1; 38.4)	0.94	0.43	(− 39.5; 40.4)	0.98
EATi	− 0.03	(− 0.9; 0.9)	0.94	− 0.6	(− 1.4; 0.2)	0.11	− 2.4	(− 7.9; 3.2)	0.39
ITFi	− 0.1	(− 0.5; 0.3)	0.58	− 0.3	(− 0.6; 0.08)	0.13	− 1.2	(− 3.4; 1.1)	0.29
VAFi	− 0.5	(− 0.9; − 0.008)	0.04	− 0.4	(− 0.9; − 0.01)	0.03	− 0.5	(− 1.0; − 0.1)	0.01
SAFi	− 0.6	(− 0.9; − 0.2)	< 0.01	− 0.3	(− 0.7; 0.2)	0.18	− 0.4	(− 0.8; 0.8)	0.11
TAFi	− 0.3	(− 0.6; − 0.1)	< 0.01	− 0.3	(− 0.5; − 0.05)	0.01	− 0.3	(− 0.49; − 0.05)	0.02

Multivariable (MV) adjustment included age, sex, body mass index, arterial hypertension, systolic blood pressure, diastolic blood pressure, diabetes mellitus dyslipidemia, and smoking status. Body mass index was not included in BMI-specific analysis

*CI* confidence interval, *EATi* epicardial adipose tissue index, *ITFi* intrathoracic fat volume index, *SAFi* subcutaneous abdominal fat index, *TAFi* total abdominal fat index, *VAFi* visceral abdominal fat index

^a^Normal weight (BMI ≤ 25 kg/m^2^) as the reference category

^b^Overweight (BMI > 25 < 30 kg/m^2^) as the reference category

To further assess if the association between body fat and AVC may vary according to the severity of the disease, we performed a stratified analysis by age-and STS-morbimortality score-groups. In patients older than 80 years, or high pre-operative risk (STS-morbimortality-score > 24%), overweight and obesity were associated with lower total AVC mass, whereas no significant association was found between BMI categories and AVC mass in patients below 80 years of age, or with STS morbimortality score ≤ 24%. Similarly, when regional body fat we observed that VAF was inversely associated with AVC mass in the elderly or high STS-morbimortality score, whereas no link between was found in low risk groups (Additional file 1: Table S2).

### Body fat and prediction of death after TAVR

Over a median follow-up of 1.2 years (Q1: 0.4; Q3: 2.7; maximum of 7.1) (97% complete), 52 patients died, 28 of them because of cardiovascular diseases-related causes. The 24 non-cardiovascular deaths were caused by sepsis (n = 11), cancer (n = 7), hip fracture (n = 4), and cerebral hemorrhage after accidental fall (n = 2).

**Fig. 2:**
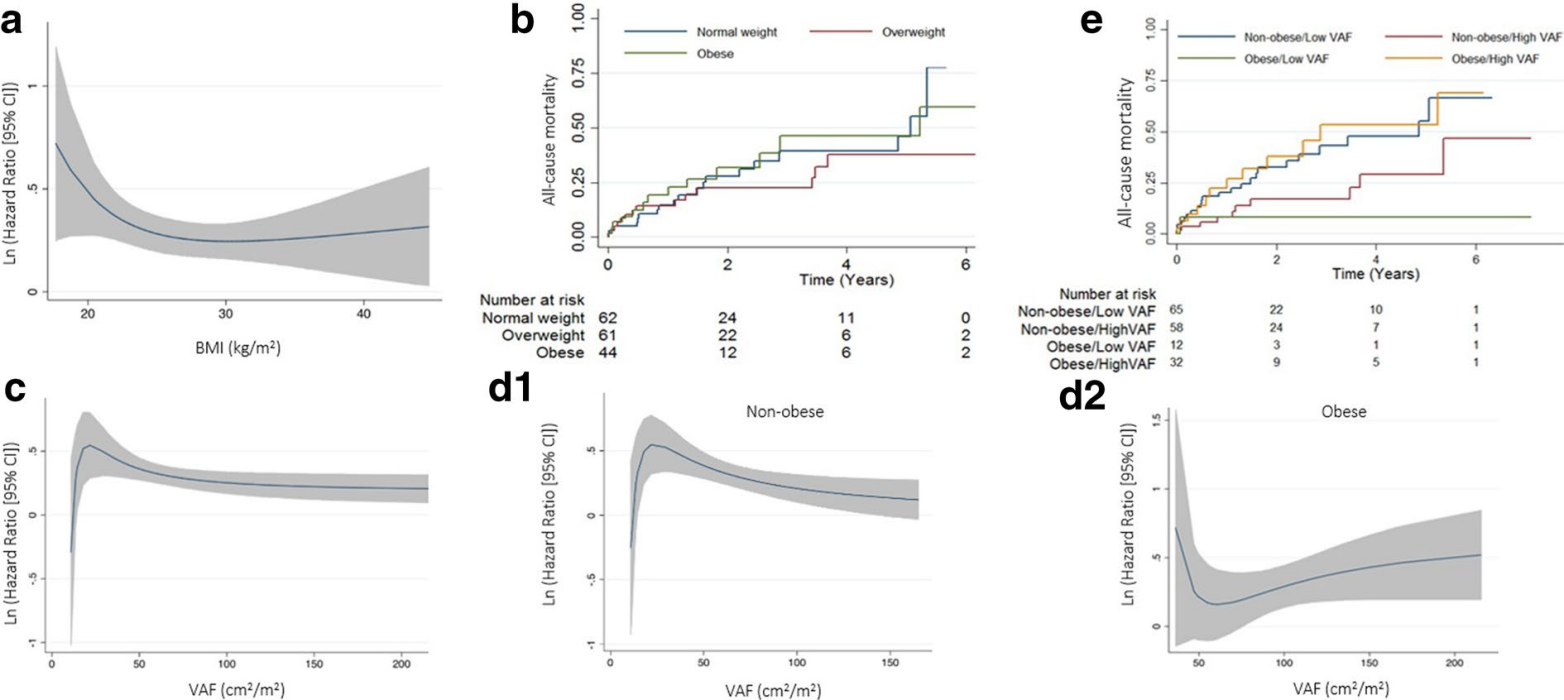
Relationship between BMI and visceral abdominal fat with all-cause mortality. Fractional polynomials analysis applied to univariate cox regression showed that the risk of all-cause mortality decreased with the increment of BMI (**a**). Kaplan–Meier curves for all-cause mortality by categories of BMI did not differ significantly but normal weight and obese patients tended to have higher incidence of death compared with the overweight (**b**). Univariate Cox regression using fractional polynomials showed an increased risk of death with the increment of VAF (**c**), but this association seemed to differ according to the patient BMI and different mortality rates curves were obtained within the non-obese (**d1**) and obese (**d2**) groups. We further combined BMI with VAF-categories, and we observed that the cumulative incidence of all-cause death was lowest in the obese that showed low amount of VAF (blue colour code) but high if VAF was high (yellow colour code); in the non-obese, low VAF identified clusters of patients at higher risk of death (**e**). *AVC* aortic valve calcification, *BMI* body mass index, *SAF* subcutaneous abdominal fat, *VAF* visceral abdominal fat

Application of a univariate Cox regression using fractional polynomials showed that the risk of all-cause mortality decreased with the increment of BMI (Fig. 2a). However, Kaplan–Meier curves for all-cause mortality by categories of BMI revealed that normal weight and obese patients tended to have higher cumulative incidence of death compared with the overweight (HR with normal weight of 1.31 [95% CI 0.65; 2.61] and HR with obese of 1.43 [95% CI 0.67; 3.06]) (Fig. 2b). In contrast, univariate Cox regression using fractional polynomials showed an increased risk of death with the increment of VAF, but this association differed according to the patient BMI (Fig. 2c): We further combined BMI with VAF-categories and different mortality rates curves were obtained within the obese and non-obese groups (Fig. 2d1, d2, respectively); at long-term follow-up, the incidence of death was significantly higher in non-obese/low VAF and in obese/high VAF patients: HR with non-obese/low VAF was 2.30 (95% confidence interval [CI] 1.12–4.91; p = 0.02) and the HR with obese/high VAF was 2.53 (95% CI 1.10–5.84; p = 0.03) compared with obese/low VAF, and did not differ with the non-obese/high VAF (Fig. 2e).

In order to explore if the association of VAF with mortality may be different according to BMI, we further investigated its predictor value by obesity groups. In non-obese, low VAF was associated with higher mortality, whereas a positive trend was apparent between VAF and all-cause death in the obese (MV-adjustment: 0.98 [95% CI (0.96; 0.99)]; p = 0.029 vs. 1.06 [95% CI (0.97; 1.10)], p = 0.385, for non-obese and obese, respectively; p value for BMI interaction of 0.04) (Table 3).

**Table 3:** Obesity-specific cox regression analysis for the association between abdominal fat with all-cause and cardiovascular-related deaths after TAVR

BMI-group	Unadjusted			Age and sex-adjusted			MV-adjusted		
	HR	95% CI	p value	HR	95% CI	p value	HR	95% CI	p value
Non-obese (< 30 kg/m^2^), n = 126									
VAFi	0.98	(0.97; 0.99)	0.021	0.98	(0.97; 0.99)	0.011	0.98	(0.96; 0.99)	0.03
SAFi	0.99	(0.98; 1.01)	0.17	0.99	(0.98; 1.0)	0.264	1.6	(0.55; 4.48)	0.39
TAFi	0.99	(0.98; 0.99)	0.021	0.99	(0.98; 0.99)	0.023	0.96	(0.93; 0.99)	0.04
VAF/SAF ratio	0.77	(0.38; 1.54)	0.455	0.64	(0.29; 1.39)	0.262	0.36	(0.12; 0.98)	0.03
Obese (BMI ≥ 30 kg/m^2^), n = 44									
VAFi	1	(0.98; 1.01)	0.91	1	(0.98; 1.01)	0.944	1.06	(0.98; 1.10)	0.38
SAFi	1	(0.98; 1.01)	0.921	0.99	(0.98; 1.01)	0.82	0.98	(0.97; 1.0)	0.76
TAFi	1	(0.98; 1.01)	0.886	0.99	(0.98; 1.01)	0.921	0.99	(0.98; 1.0)	0.30
VAF/SAF ratio	1.04	(0.31; 3.53)	0.949	1.6	(0.27; 9.56)	0.604	30.2	(0.03; 300.1)	0.14

Multivariate (MV) adjustment included sex, age, systolic blood pressure, diastolic blood pressure, arterial hypertension, dyslipidemia, diabetes mellitus, smoking status, peripheral arterial disease, chronic obstructive pulmonary disease, neoplasia, atrial fibrillation, New York Heart Association class, mean aortic valve gradient, left ventricular ejection fraction

*CI* confidence interval, *HR* hazard ratio, *VAFi* visceral abdominal fat index, *SAFi* subcutaneous abdominal fat index, *TAFi* total abdominal fat index

## Discussion

In the present study, we determined the association of global (reflected by the BMI) and regional adiposity (measured by computed tomography) with AVC and mortality in a cohort of elderly AS patients who underwent TAVR. Our main findings were: (1) higher AVC mass was associated with low BMI and VAF (2) low BMI was associated with higher incidence of death, but (3) the association of VAF with mortality differed according to BMI: low VAF was associated with increased mortality in non-obese, while a positive trend were found in the obese.

Previous studies have shown that metabolic syndrome and diabetes are not only associated with increased prevalence of computed tomography-detected AVC [21], but also with increased progression of AS [4, 5], increased likelihood of incident AVC [4] and accelerated degeneration of bioprosthetic aortic valve [22]. Paradoxically, we demonstrated an inverse association of BMI with AVC but these associations were present only in the older and higher pre-intervention risk patients. Importantly, previous studies investigating the association between metabolic syndrome and AVC included younger subjects and less severe AS patients (moderate AS in low-intermediate cardiovascular risk patients) than we did in our study. In the ASTRONOMER (Aortic Stenosis Progression Observation: Measuring Effects of Rosuvastatin) trial, Capoulade et al. [5] showed that metabolic syndrome is, actually, an independent predictor of AS progression but their results suggested that the pathophysiological mechanisms leading to the development and progression of AS may be different between the older and younger; in younger subjects (< 57 years of age), the rate of AS progression was twofold faster in subjects with metabolic syndrome than those without metabolic syndrome, whereas AS progression was similar in older subjects with or without metabolic syndrome. Considering these data, a neutral effect, instead of a negative association, would be expected in our study. But, likewise ours findings, Rogge et al. [7] in a sub-analysis of the SEAS (Simvastatin Ezetimibe in Aortic Stenosis) trial, reported lower rates of ischemic events and AS-related events in overweight compared with normal weight patients, with similar incidence of events between obese and normal weigh patients. However, when age was added to the multivariable Cox regression model, the association of overweight with lower cardiovascular events did not remain significant, suggesting this association is partially explained by the ageing-related body fat changes.

Confounding by smoking and reverse causality have been appointed as possible explanations for the obesity paradox which has been considered by some authors as a statistical artifact arising from biases in observational studies. In the present study, the distributions of current and former smokers did not differ between BMI-categories and we further adjusted our models for smoking status (and also chronic obstructive pulmonary disease). Another concern is reverse causation due to age and disease-induced weight loss. After the age of 60 years, body weight on average tends to decrease. Ageing is generally associated with increased total adiposity over the adult lifespan, until extreme old age when fat mass decreases. The consequences of this fat mass loss are not well understood, but it may be an important indicator of global deterioration in health [16, 23]. Moreover, previous studies in patients with congestive heart failure showed a strong continuous relationship between weight loss and increased mortality. In a post-doc analysis of the SOLVD trial, Anker et al. [24] were the first to suggest that any weigh loss, independent of the patients’ weight at baseline, is related to poor survival. More recently, Rossignol et al. [25] observed significant weight loss in 16.4 and 15.7% of patients from the GISSI-HF and Val-HeFT studies, respectively, which corresponded to an increased risk of death of 20% (GISSI-HF) to 150% (Val-HeFT). Mechanically, the weight loss-related to heart failure has been explained as part of its metabolic impairment, namely insulin resistance, global anabolic blunting and catabolic overactivity due to catecholamine, TNF-alpha, and natriuretic peptides release. As a result, the heart failure metabolic phenotype is characterized by body tissues wasting including muscle, fat, and bone leading to total weight loss, and ultimately to the development of cachexia [26–28]. Hence, the favorable prognosis of obese patients with heart failure might reflect that they have higher metabolic reserves allowing them to better tolerate the catabolic stress than the non-obese. However, the occurrence of unintentional weight loss may be a surrogate for disease progression even in the obese patients [29].

Even though, the obesity paradox can only be clarified through longitudinal analysis of how to changes in body composition affect the incidence of mortality over time, it is important to note that studies supporting its existence almost always use BMI as the only measure of obesity. However, because BMI is an imperfect method of measuring obesity that does not capture differences regarding the regional body fat distribution and body composition, some studies using other meaures of obesity such as waist circumference and waist to hip ratio render showed conflictutual results [30].

The individual contribution of visceral fat to mortality in patients with AS has not been explored yet. Since, computed tomography is part of the routine evaluation before TAVR as a noninvasive method to plan the intervention (i.e. assessing vascular accesses, measuring aortic annulus anatomy etc.), information on thoracic and abdominal body fat distribution is routinely available in these patients. In this study, we explored how visceral thoracic and abdominal fat relates to AVC and with all-cause and cardiovascular related-causes of deaths. First, we showed that VAF was inversely associated with AVC mass score, suggesting that the obesity paradox may also be observed in the visceral fat compartment. Secondly, we verified a positive association between VAF and mortality but different mortality rates were seem according to the patients BMI: low VAF predicted mortality in the non-obese but the opposite seemed to occur in the obese.

Visceral fat quantification may offer an opportunity to identify when patients have reached a clinically state at which the mortality risk is increased. In 61 patients with advanced heart failure with severely dysfunctional left ventricles. Chokshi et al. [31], interestingly, showed that the systemic and local metabolic derangements improved after long-term left-ventricular assistance device support leading to reduced myocardial levels of toxic lipid intermediates and improved cardiac insulin signaling. This finding indicates that point reversibility may possibly exist in the heart failure related-metabolic dysfunction and that treatment interventions can modify the pathophysiological cascade of the disease.

### Study limitations and strengths

In this study, we determined the association between baseline BMI and long-term mortality but we did not record neither past patients’ weight (i.e. weight at youth- and middle-ages), nor the patient weight after intervention, and thus the impact of weight changes over time on mortality could not be assessed. Furthermore, rather than using computed tomography to quantify body fat compartment a more sensitive characterization of body fat distribution and body composition could be obtained by X-ray densitometry or bioelectrical impedance analysis; hence, we were unable to study the association of fat mass and fat-free mass on mortality. We also acknowledge that TAVR procedure per se and the management of patients after the intervention had changed over the last decade. Considering this is a small sample monocentric study, we cannot exclude that technical issues have affected our findings. Similarly, the fact that only 44 patients were obese could have limited our statistical power and explain why we did find a borderline positive association between VAF and mortality.

## Conclusions

In elderly AS patients submitted to TAVR, low BMI were paradoxically associated with AVC and mortality, and VAF may identify patients at different risk of death in the non-obese. The present findings may have implications on the understanding of obesity-mortality relations and suggest that VAF quantification in the pre-intervention assessment of patients referred to TAVR may help to identify the pre-cachexia (“at risk”) phase when the cardiovascular intervention can potentially reverse the heart failure-induced metabolic syndrome.

## What is known about the topic?

Previous studies showed that metabolic syndrome is associated with AVC, progression of AS and accelerated degeneration of bioprosthetic aortic valve. However, the available data on patients after TAVR is still scarce, and the role of fat distribution assessment in this context has not yet been established.

## What does this study add?

In elderly AS patients submitted to TAVR, low BMI was associated with AVC and mortality. Pre-intervention assessment of abdominal fat distribution by computed tomography may help a better discriminator of patients-risk when used in combination with BMI than BMI alone.

## Additional file

**Additional file 1: Table S1.** Intraclass correlation coefficients (ICC) for inter and intrarater reliability of fat measurements. **Table S2.** Age- and STS morbimortality score-specific univariable and multivariable median regression analyses for the association of obesity and abdominal fat with total AVC mass.

## Abbreviations

AS: aortic stenosis
AVC: aortic valve calcification
BMI: body mass index
TAVR: transcatheter aortic valve replacement
VAF: visceral abdominal fat
SAF: subcutaneous abdominal fat
