# Supplementary material for: Association of body mass index and visceral fat with aortic valve calcification and mortality after transcatheter aortic valve replacement: the obesity paradox in severe aortic stenosis
Source: Diabetol Metab Syndr. 2017 Oct 19;9:86. doi: 10.1186/s13098-017-0285-2 (PMC5649084; doi:10.1186/s13098-017-0285-2)
Supplement: Supplementary file 1 — Additional file 1: Table S1. Intraclass correlation coefficients (ICC) for inter and intrarater reliability of fat measurements. Table S2. Age- and STS morbimortality score-specific univariable and multivariable median regression analyses for the association of obesity and abdominal fat with total AVC mass. [file 13098_2017_285_MOESM1_ESM.docx]

**Additional Tables**

**Additional Table** S1 – Intraclass correlation coefficients (ICC) for inter and intrarater reliability of fat measurements.

**Additional Table S2** – Age-, and STS morbimortality score-specific univariable and multivariable quantile regression analyses for the association of obesity and abdominal fat with total AVC mass.

| \| **Additional Table S1** – Intraclass correlation coefficients (ICC) for inter and intrarater reliability of fat measurements (n=55) \| \| \| \| --- \| --- \| --- \| \| **Fat measure** \| **Intrarater ICC**  **(95% CI)** \| **Interrater ICC**  **(95% CI)** \| \| Epicardial fat \| 0.98 (0.90-0.99) \| 0.96 (0.82-0.99) \| \| Total intrathoracic fat \| 0.92 (0.67-0.98) \| 0.91 (0.60-0.98) \| \| Total abdominal fat \| 0.98 (0.93-0.99) \| 0.97 (0.91-0.99) \| \| Visceral abdominal fat \| 0.95 (0.94-0.99) \| 0.96 (0.90-0.99) \|   CI=confidence interval, ICC=intraclass correlation coefficient |
| --- | --- | --- | --- | --- | --- | --- | --- | --- | --- | --- | --- | --- | --- | --- | --- | --- | --- | --- |

| **Additional Table S2** – Age, coronary artery disease and STS morbimortality score-specific univariable and multivariable quantile regression analyses for the association of obesity and abdominal fat with total AVC mass | | | | | | | | | | | | | |  |
| --- | --- | --- | --- | --- | --- | --- | --- | --- | --- | --- | --- | --- | --- | --- |
|  | **Age≥80 yrs** | | | **Age<80 yrs** | | | **STS morbimortality >24%** | | | | **STS morbimortality ≤24%** | | |  |
|  | **Beta** | **95% CI** | **p value** | **Beta** | **95% CI** | **p value** | **Beta** | **95% CI** | **p value** | | **Beta** | **95% CI** | **p value** | |
| **Overweight*** |  |  |  |  |  |  |  |  | |  |  |  |  | |
| Unadjusted | -76.7 | (-127.8; -25.6) | <0.01 | -54.5 | (-129.6; 20.6) | 0.15 | -47.2 | (-96.5; 20.1) | | 0.06 | -83.2 | (-138.7; -27.6) | <0.01 | |
| Age and sex-adjusted | -69.5 | (-114.9; -24.0) | <0.01 | -23.2 | (-86.5; 40.1) | 0.46 | -25.4 | (-71.1; 20.3) | | 0.27 | -53.7 | (-117.1; 9.6) | 0.09 | |
| MV-adjusted | -44.3 | (-92.5; 3.9) | 0.07 | -32.2 | (-93.2; 29.2) | 0.29 | -12.1 | (-20.2; -8.3) | | 0.03 | -42.8 | (-50.8; 4.3) | 0.07 | |
| **Obese*** |  |  |  |  |  |  |  |  | |  |  |  |  | |
| Unadjusted | -77.5 | (-140; -14.4) | 0.02 | -48.6 | (-122.0; 24.8) | 0.19 | -48.7 | (-100.4; 30.4) | | 0.06 | -81.2 | (-144.2; -18.1) | 0.01 | |
| Age and sex-adjusted | -69.3 | (-126.5; -12.0) | 0.01 | -23.1 | (-86.0; 40.6) | 0.73 | -19.2 | (-69.3; 30.9) | | 0.44 | -67.3 | (-140.6; 6.02) | 0.07 | |
| MV-adjusted | -38.9 | (-101.2; 23.4) | 0.21 | -35.3 | (-101.0; 30.8) | 0.29 | -32.6 | (-77.9; 12.7) | | 0.15 | -74.3 | (-164; 15.8) | 0.10 | |
| **VAFi** |  |  |  |  |  |  |  |  | |  |  |  |  | |
| Unadjusted | -0.5 | (-1.0; 0.05) | 0.07 | -0.5 | (-1.3; 0.3) | 0.22 | -0.6 | (-1.1; -0.007) | | 0.04 | -0.2 | (-1.0; 0.7) | 0.68 | |
| Age and sex-adjusted | -0.5 | (-1.1; 0.03) | 0.06 | -0.4 | (-1.1; 0.4) | 0.35 | -0.5 | (-1.0; -0.04) | | 0.03 | -0.3 | (-1.1; 0.5) | 0.40 | |
| MV-adjusted | -0.4 | (-1.3; -0.04) | 0.03 | -0.4 | (-1.0; 0.2) | 0.19 | -0.6 | (-1.1; -0.02) | | 0.04 | -0.6 | (-1.4; 0.2) | 0.14 | |
| **SAFi** |  |  |  |  |  |  |  |  | |  |  |  |  | |
| Unadjusted | -0.5 | (-0.9; 0.01) | 0.05 | -0.6 | (-1.4; 0.1) | 0.12 | -0.6 | (-0.9; -0.2) | | <0.01 | -0.6 | (-1.3; 0.1) | 0.11 | |
| Age and sex-adjusted | -0.8 | (-0.9; 0.6) | 0.64 | -0.4 | (-0.9; 0.2) | 0.20 | -0.3 | (-0.8; 0.2) | | 0.19 | -0.5 | (-1.2; 0.3) | 0.21 | |
| MV-adjusted | -0.2 | (-0.8; 0.4) | 0.51 | -0.4 | (-1.3; 0.4) | 0.28 | -0.3 | (-0.9; 0.3) | | 0.32 | -0.1 | (-0.9; 0.6) | 0.72 | |
| **TAFi** |  |  |  |  |  |  |  |  | |  |  |  |  | |
| Unadjusted | -0.4 | (-0.8; 0.1) | <0.01 | -0.3 | (-0.7; 0.02) | 0.04 | -0.5 | (-0.7; -0.2) | | <0.01 | -0.2 | (-0.6; 0.2) | 0.43 | |
| Age and sex-adjusted | -0.3 | (-0.6; -0.01) | 0.03 | -0.1 | (-0.4; 0.2) | 0.43 | -0.4 | (-0.7; -0.1) | | <0.01 | -0.2 | (-0.6; 0.2) | 0.31 | |
| MV-adjusted | -0.4 | (-0.8; -0.08) | 0.0 | -0.3 | (-0.6; 0.06) | 0.10 | -0.4 | (-0.8; -0.05) | | 0.02 | -0.1 | (-0.5; 0.4) | 0.82 | |
| CI= confidence interval, SAFi=subcutaneous abdominal fat index, STS= Society of Thoracic Surgeons, TAFi= total abdominal fat index, VAFi= visceral abdominal fat index* Normal weight (BMI≤25 kg/m2) as the reference category. Multivariable (MV) adjustment included age, sex, body mass index, arterial hypertension, systolic blood pressure, diastolic blood pressure, diabetes, and dyslipidemia. Body mass index not included in BMI-specific analysis | | | | | | | | | | | | | |  |
